# Imbalanced effector and regulatory cytokine responses may underlie mycobacterial immune restoration disease

**DOI:** 10.1186/1742-6405-5-9

**Published:** 2008-04-29

**Authors:** Andrew Lim, Lloyd D'Orsogna, Patricia Price, Martyn A French

**Affiliations:** 1 School of Pathology and Laboratory Medicine, University of Western Australia, Level 2 Medical Research Foundation, Rear 50 Murray Street, Perth 6000, Australia; 2 Department of Clinical Immunology, Sir Charles Gairdner Hospital, Hospital Avenue, Nedlands 6009, Australia; 3 Department of Clinical Immunology and Immunogenetics, Level 2 North Block, Royal Perth Hospital, Wellington Street, Perth 6000, Australia

## Abstract

**Background:**

Immune restoration disease (IRD) is an adverse consequence of antiretroviral therapy, where the restored pathogen-specific response causes immunopathology. Mycobacteria are the pathogens that most frequently provoke IRD and mycobacterial IRD is a common cause of morbidity in HIV-infected patients co-infected with mycobacteria. We hypothesised that the excessive effector immune response in mycobacterial IRD reflects impaired regulation by IL-10.

**Results:**

We studied two patients who experienced mycobacterial IRD during ART. One patient developed a second episode of IRD with distinct clinical characteristics. Findings were compared with patients 'at risk' of developing IRD who had uneventful immune recovery. Peripheral blood mononuclear cells (PBMC) from all subjects were stimulated with mycobacterial antigens in the form of purified protein derivative (PPD). Supernatants were assayed for IFNγ and IL-10. In response to PPD, PBMC from IRD patients generated IFNγ during the first IRD episode, whilst cells from non-IRD controls produced more IL-10.

**Conclusion:**

We present preliminary data from two HIV-infected patients showing an imbalance between IFNγ and IL-10 responses to mycobacterial antigens during mycobacterial IRD. Our findings suggest that imbalanced effector and regulatory cytokine responses should be investigated as a cause of IRD.

## Background

Immune restoration disease (IRD) after commencing antiretroviral therapy (ART) is considered to be a consequence of restoring an immune response against an active (often quiescent) infection by an opportunistic pathogen, or antigens of non-viable pathogens, that results in immunopathology [1,2]. *Mycobacterium tuberculosis *(*Mtb*) and non-tuberculous mycobacteria are pathogens that commonly provoke IRD [3,4]. Patients experiencing mycobacterial IRD often present with fever and lymphadenitis, but may also have pulmonary infiltrates or inflammatory masses. A cutaneous delayed-type hypersensitivity (DTH) response to mycobacterial antigens is a characteristic finding [1,5,6], and coincides with excessive production of Type 1 (Th1) cytokines [7], probably by memory CD4^+ ^T cells. However, the immunopathogenesis of mycobacterial IRD is not fully understood and may be variable since the clinical presentations can be diverse. Tissue inflammation presenting during the first few months of ART usually has the features of a DTH immune response [2], but some patients present with suppurating lymphadenitis and/or disease that presents later [8-10].

Immunity to mycobacterial infections is influenced by the counteracting effects of effector cytokines and regulatory cytokines, such as interleukin (IL)-10, in patients with and without HIV infection [11-14]. As IL-10 provides a regulatory mechanism for Th1 memory CD4^+ ^T cell responses [15,16], we hypothesised that the excessive effector response in mycobacterial IRD reflects impaired regulation by IL-10. We present preliminary data from two HIV-infected patients showing an imbalance between interferon-gamma (IFNγ) and IL-10 responses to mycobacterial antigens during mycobacterial IRD.

## Results

### Subjects

Patient 1 (P1) was a 48-year-old Caucasian male who developed *Mycobacterium celatum *IRD one month after commencing ART. This case has been described previously [17]. Briefly, he presented with a CD4^+ ^T cell count of 48/μL and plasma HIV RNA level >100,000 copies/mL. He developed fever on day 11 of ART and sputum collected on each of the following 3 days yielded *M. celatum*. Chest radiography and a computer tomography (CT) scan revealed areas of consolidation in both lungs. On day 27 of ART his CD4^+ ^T cell count had increased to 189/μL, his plasma HIV RNA level had decreased to 933 cells/μL and DTH skin testing demonstrated an 18 mm response to purified protein derivative (PPD) and a 10 mm response to *Mycobacterium avium *antigen. Prednisone was administered and the patient's symptoms resolved completely.

Patient 2 (P2) was a 51-year-old Caucasian male who presented with a CD4^+ ^T cell count of 16/μL and plasma HIV RNA level of 54,954 copies/mL. Two routine blood cultures taken at initial presentation grew *Mycobacterium avium *complex (MAC). He was treated with ethambutol, rifabutin and azithromycin. DTH skin tests with *M. avium *antigen and PPD both gave reactions of 0 mm, demonstrating anergy towards mycobacterial antigens.

Six weeks after initial presentation, he commenced ART consisting of efavirenz, zidovudine and lamivudine. Four months later, he was seen for a routine assessment and had no specific complaints. However, a right axillary lymph node was detected and the liver was palpable. Blood tests revealed a haemoglobin level of 99 g/L, serum CRP level of 51 mg/L and ESR of 100 mm/hr but normal LDH and liver function tests. His CD4^+ ^T cell count was 42/μL and plasma HIV RNA level <50 copies/mL. An aspirate of the right axillary lymph node and a sputum sample both grew MAC. Blood cultures were negative. A chest CT scan revealed marked axillary and mediastinal lymphadenopathy. On this occasion, DTH skin testing gave reactions of 18 mm to *M. avium *antigen and 17 mm to PPD. He continued on anti-MAC antibiotics and commenced prednisolone therapy for likely MAC IRD. After 4 weeks the lymphadenopathy was resolving and CRP was normal. Plasma HIV RNA remained undetectable.

He had an uneventful course for 6 months until he represented unwell with 10 kg weight loss and night sweats. There was a 5 × 6 cm draining lymph node in the cervical region and a chronic discharging sinus in the right axilla. No other lymphadenopathy or organomegaly was palpable. Plasma HIV RNA was still undetectable; however the CD4^+ ^T cell count was only 42/μL. A surgically resected lymph node revealed the presence of mycobacteria, but these could not be cultured. A granulomatous inflammatory response with necrosis, neutrophils and karryorhectic debris (dead neutrophils) at the centre of necrotic areas was also demonstrated. Tests for *Mtb *DNA in the lymph node, blood cultures for mycobacterial infection and a whole blood IFN-γ release assay with *Mtb*-specific antigens (QuantiFERON-TB Gold, Cellestis) were all negative. The serum CRP level was normal. DTH skin testing revealed an absent response to *M. avium *antigen and only a 7 mm reaction to PPD. He continued anti-MAC antibiotics and recommenced prednisolone therapy.

### Cytokine production

**Figure 1:**
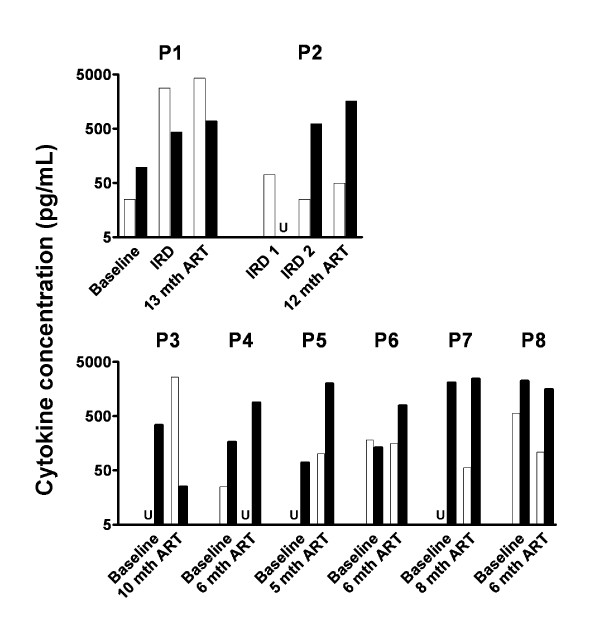
**More IFNγ than IL-10 is produced during an IRD**. PBMC from two IRD patients (P1 and P2) and six non-IRD patients (P3 to P8) were stimulated with PPD for 24 hours. Concentrations of IFNγ (white bars) and IL-10 (black bars) were measured in culture supernatants. Baseline refers to a time-point immediately prior to commencement of ART. U, undetectable.

**Figure 2:**
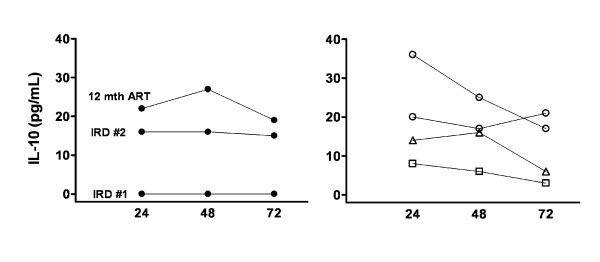
**Deficient IL-10 production by PBMC stimulated with PPD from P2 during his first IRD episode**. IL-10 production was assessed over 72 hours in PBMC from P2 (filled circles; left plot) and compared with responses of four other PPD-responsive individuals (open circles, two uninfected donors; triangles, HIV-negative patient with active tuberculosis; squares, P3 after 16 months of ART; right plot). P3 had a history of treated tuberculosis many years earlier. mth, months.

To examine the production of effector and regulatory cytokines, peripheral blood mononuclear cells (PBMC) from both IRD patients and from six non-IRD patients were cultured with antigens and a mitogen control and supernatants assayed for IFNγ and IL-10. During the first episodes of IRD, PBMC from P1 and P2 produced more IFNγ than IL-10 in response to PPD (Figure 1). P1 retained higher IFNγ production 12 months later. Undetectable levels of IL-10 from P2 were confirmed in a second culture over 72 hours (Figure 2). In contrast, PBMC from 5 of the 6 non-IRD patients produced more IL-10 than IFNγ after 6 months of ART (Figure 1). P3 had a history of treated tuberculosis many years earlier and was the only non-IRD patient to exhibit higher production of IFNγ than IL-10.

P1 experienced disseminated CMV disease prior to ART. To demonstrate that high levels of PPD-induced IFNγ production during his IRD was specific for the provoking pathogen, his PBMC were stimulated with CMV antigen. At the time of IRD, IFNγ was undetectable (<15 pg/mL) in the supernatants of CMV-stimulated cultures.

The second episode of IRD in P2 had different immunological characteristics to the first episode. IFNγ was not detected in supernatants of PBMC stimulated with PPD (Figure 1). However, IL-10 was detectable after 24, 48 or 72 hours stimulation (Figure 2). These IL-10 responses remained steady after 12 months on ART.

## Discussion

We present clinical and laboratory data from two patients with mycobacterial IRD, and appropriate controls, that support our hypothesis that IRD might reflect failure to produce sufficient IL-10 during restoration of mycobacteria-specific effector CD4^+ ^T cell responses after commencing ART. We used PPD as an antigen to investigate pathogen-specific cytokine responses because we and others have shown that IRD associated with infection by *Mtb *and non-tuberculous mycobacteria (NTM) is associated with increased cellular immune responses to PPD [1,2,5,7,17]. Our controls were considered to be 'at risk' of developing IRD because we have previously shown that 'subclinical' infection with NTM is almost inevitable when HIV patients in our population are severely immunodeficient [18].

PPD-stimulated PBMC from both IRD patients produced less IL-10 than IFNγ during the first episode of IRD. High IFNγ production has been previously demonstrated in *Mtb *IRD [7]. In contrast, IL-10 production by PPD-stimulated PBMC predominated in donors without IRD, suggesting sufficient regulatory control. The more vigorous IFNγ response in P1 relative to P2 may reflect better immune reconstitution as P1 experienced an approximately 4-fold increase in CD4^+ ^T cell count after only 1 month of ART, while P2 had a persistently low CD4^+ ^T cell count even after 10 months of ART. However, both patients displayed strong DTH skin test responses at the time of IRD, indicating that restoration of cellular immune function may not correlate with circulating CD4^+ ^T cell counts. Higher production of IFNγ relative to IL-10 in P3 may reflect *Mtb*-specific immunological memory as a consequence of previous tuberculosis.

We were able to study two episodes of IRD in P2. His second IRD episode had different immunological and clinical characteristics to the first episode. A cutaneous DTH response to *M. avium *antigen was absent. High IL-10 and low IFNγ production were demonstrated following PPD stimulation of PBMC. With the clinical findings of sinus formation from lymph nodes and predominance of neutrophils in the lymph node biopsy, our data suggests that this episode did not fit the "classical" Th1 presentation of mycobacterial IRD. Investigation of other effector T cells might be informative, particularly Th17 cells as these cells may promote neutrophil responses [19].

## Conclusion

In conclusion, we suggest that future studies of the immunopathogenesis of mycobacterial IRD should include analyses of mycobacteria-specific effector and regulatory cytokine responses.

## Methods

### Laboratory methods and controls

PBMC were cryopreserved prior to ART (P1 only), at each IRD episode and after 12 months on ART. Six male HIV-infected patients who did not develop IRD after commencing ART (non-IRD patients, P3 to P8) were sampled prior to ART and approximately 6 months later. All non-IRD patients had CD4^+ ^T cell counts below 100/μL and plasma HIV RNA levels >4 log_10 _copies/mL before ART. At the second PBMC collection, 5 non-IRD patients had achieved and maintained plasma HIV RNA levels of <50 copies/mL, while the remaining patient had achieved and maintained plasma HIV RNA levels >2 logs below his baseline level. Four non-IRD patients experienced at least a four-fold increase in their CD4^+ ^T cell count after 6 months of ART (total CD4^+ ^T cell count increased to above 150/μL), while the other 2 patients increased their CD4^+ ^T cell counts less than three-fold (total CD4^+ ^T cell count remained below 100/μL). Non-IRD patient 3 (P3) had a history of treated tuberculosis several years earlier.

PBMC were isolated by Ficoll separation of heparinised whole blood and cryopreserved in RPMI with 10% dimethylsulfoxide. Thawed PBMC were cultured in 10% FCS/RPMI at 2.5 × 10^6 ^cells/mL with 10 μg/mL PPD (Statens Serum Institute) or CMV antigen (AD169 lysate), and 12.5 μg/mL phytohaemaggutinin (mitogen control) at 37°C for 24 hours. Supernatants were assayed by ELISA for IFNγ (BD Biosciences) and IL-10 (R&D Systems). Unstimulated cells were cultured in parallel.

In a second experiment, PBMC from P2, P3, a HIV-negative patient with active tuberculosis and two uninfected donors were cultured at 1 × 10^6 ^cells/mL with PPD over a 72-hour time-course. Both uninfected donors were known to have PPD-specific cells detectable by ELISpot. Supernatants were assayed for IL-10 at 24-hour intervals by cytometric bead array (BD Biosciences).

Informed consent was obtained from all individuals and the study was approved by the Ethics Committee of Royal Perth Hospital.

## Competing interests

The authors declare that they have no competing interests.

## Authors' contributions

AL carried out the labwork and drafted the manuscript. LD prepared the clinical reports for the IRD patients. PP helped to draft the manuscript. MAF conceived and designed the study, and helped draft the manuscript. All authors read and approved the final manuscript.
